# The role of autophagy in colitis-associated colorectal cancer

**DOI:** 10.1038/s41392-018-0031-8

**Published:** 2018-11-30

**Authors:** Yuhui Wu, Junlin Yao, Jiansheng Xie, Zhen Liu, Yubin Zhou, Hongming Pan, Weidong Han

**Affiliations:** 1 Department of Medical Oncology, Sir Run Run Shaw Hospital, College of Medicine, Zhejiang University, Hangzhou, 310016 Zhejiang, China; 2 Laboratory of Cancer Biology, Institute of Clinical Science, Sir Run Run Shaw Hospital, College of Medicine, Zhejiang University, Hangzhou, 310016 Zhejiang, China; 3 Center for Translational Cancer Research, Institute of Biosciences and Technology, Texas A&M University Health Science Center, Houston, TX 77030 USA

## Abstract

Autophagy is an evolutionarily conserved catabolic process that eliminates harmful components through lysosomal degradation. In addition to its role in maintaining cellular homeostasis, autophagy is critical to pathological processes, such as inflammation and cancer. Colitis-associated colorectal cancer (CAC) is a specific type of colorectal cancer that develops from long-standing colitis in inflammatory bowel disease (IBD) patients. Accumulating evidence indicates that autophagy of microenvironmental cells plays different but vital roles during tumorigenesis and CAC development. Herein, after summarizing the recent advances in understanding the role of autophagy in regulating the tumor microenvironment during different CAC stages, we draw the following conclusions: autophagy in intestinal epithelial cells inhibits colitis and CAC initiation but promotes CAC progression; autophagy in macrophages inhibits colitis, but its function on CAC is currently unclear; autophagy in neutrophils and cancer-associated fibroblasts (CAFs) promotes both colitis and CAC; autophagy in dendritic cells (DCs) and T cells represses both colitis and CAC; autophagy in natural killer cells (NKs) inhibits colitis, but promotes CAC; and autophagy in endothelial cells plays a controversial role in colitis and CAC. Understanding the role of autophagy in specific compartments of the tumor microenvironment during different stages of CAC may provide insight into malignant transformation, tumor progression, and combination therapy strategies for CAC.

## On colon ‘cleansing’ and cancer care

A cellular recycling process called autophagy plays a prominent but complicated role in the natural history of inflammation-induced colorectal cancer. Autophagy helps cells to break down unnecessary or unwanted components, but can also eliminate harmful biomolecules and even pathogens. Researchers led by Hongming Pan and Weidong Han of Zhejiang University in China have reviewed the involvement of this process in colorectal tumorigenesis in the aftermath of inflammatory conditions like ulcerative colitis, and highlight the challenges of targeting autophagy therapeutically. For example, autophagy in epithelial cells can protect against cancer onset by eliminating inflammation-promoting compounds and signals. However, once an epithelial tumor forms, autophagy can have the unwanted effect of helping tumors thrive and overcome therapy. As such, autophagy-modulating agents must be carefully selected in terms of when and how they are employed for cancer treatment.

## Introduction

Autophagy is an evolutionarily conserved cellular process by which cells digest their unneeded intracellular contents via lysosomal degradation and recycle the basic components, resulting in the maintenance of cell survival during stress responses. For example, autophagy enables the cell to adapt its metabolism and meet its energy needs during starvation by degrading and recycling proteins, lipids, and carbohydrates. Additionally, autophagy is essential for preventing inflammation and cancer through clearance of disease-causing aggregated proteins, damaged DNA and pathogenic bacteria.^1^ There are three types of autophagy: microautophagy, chaperone-mediated autophagy (CMA), and macroautophagy. In this review, we focus on macroautophagy, which is generally referred to as “autophagy”.

**Fig. 1:**
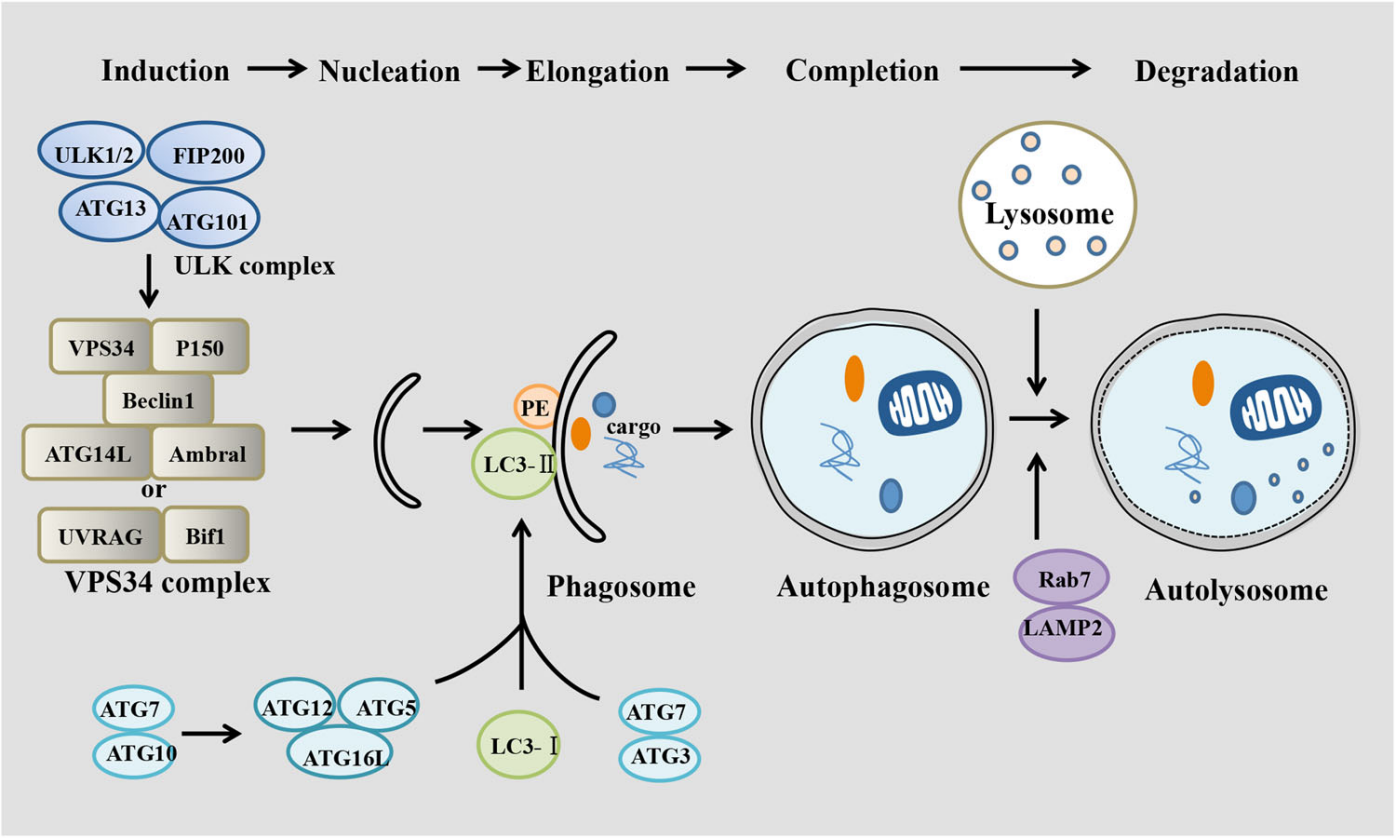
Different stages of autophagy. Autophagy includes several steps: induction, nucleation, elongation, completion, and degradation. Activated mTOR depresses the “protein kinase autophagy regulatory complex” that includes ULK1/2, Atg13, FIP200, and Atg101. This complex activates the “autophagic core complexes” including the VPS34-Beclin1-Ambra1-Atg14L-P150 complex and the VPS34-Beclin1-UVRAG-Bif1-P150 complex, which activate phagophore formation. Atg7 and Atg10 help ATG16L1 form a complex with ATG5 and ATG12, which multimerizes and then lipidates LC3-I into LC3-II. Atg7, and Atg3 mediate LC3-II conjugating to PE. Then, the phagophore recruits cargo and closes to form the autophagosome, which fuses with a lysosome to form the autophagolysosome with the help of LAMP2 and Rab7, leading to degradation of cargo as well as the inner membrane

Autophagy is a precisely controlled multi-layered system, mainly containing five stages: initiation (induction), phagophore nucleation, autophagosome formation (elongation), lysosome fusion (completion), and finally degradation.^2^ Initiation begins with activation of the Unc-51-like kinases 1 (ULK1) complex, including ULK1/2, ATG13, FIP200, and ATG101. Subsequently, the class III PI3K complex (comprising VPS34, Beclin1, UVRAG, Bif1, and P150) is activated by the ULK1 complex, which promotes phagophore nucleation. The ATG5–ATG12 complex conjugates with ATG16 to expand the autophagosome membrane, LC3-I is conjugated with phosphatidylethanolamine (PE) to form LC3II, and LC3-II is then attached to the phagophoresome membrane, recruiting cargo, and completing autophagosome formation. Ultimately, the autophagosome fuses with a lysosome, forming the autolysosome. Lysosome fusion leads to the breakdown of phagocytosed cellular components by exposing them to acidic hydrolases. Damaged or unwanted proteins are degraded into amino acids and then transported into the cytoplasm for reuse (Fig. 1).

Chronic inflammation is a high-risk factor for cancer. It is well known that patients with inflammatory bowel disease (IBD), including Crohn’s disease (CD), and ulcerative colitis (UC), have two- to three times increased risk of developing colorectal cancer (CRC) compared with healthy people, and this cancer is known as “colitis-associated colorectal cancer (CAC)”.^3^ CAC develops from nonneoplastic inflammatory epithelium that progresses to cancer. Inflammation induces robust genotoxic responses, such as DNA damage and mutations to vital genes (*p53, c-src, k-ras, β-catenin*, and *APC*), which subsequently drives CAC initiation in IBD patients. In addition, inflammation activates the signal transducer and activator of transcription 3 (STAT3) and β-catenin signaling pathways, which induce proliferation and remodeling of epithelial cells and then promote tumor development.^4,5^

**Fig. 2:**
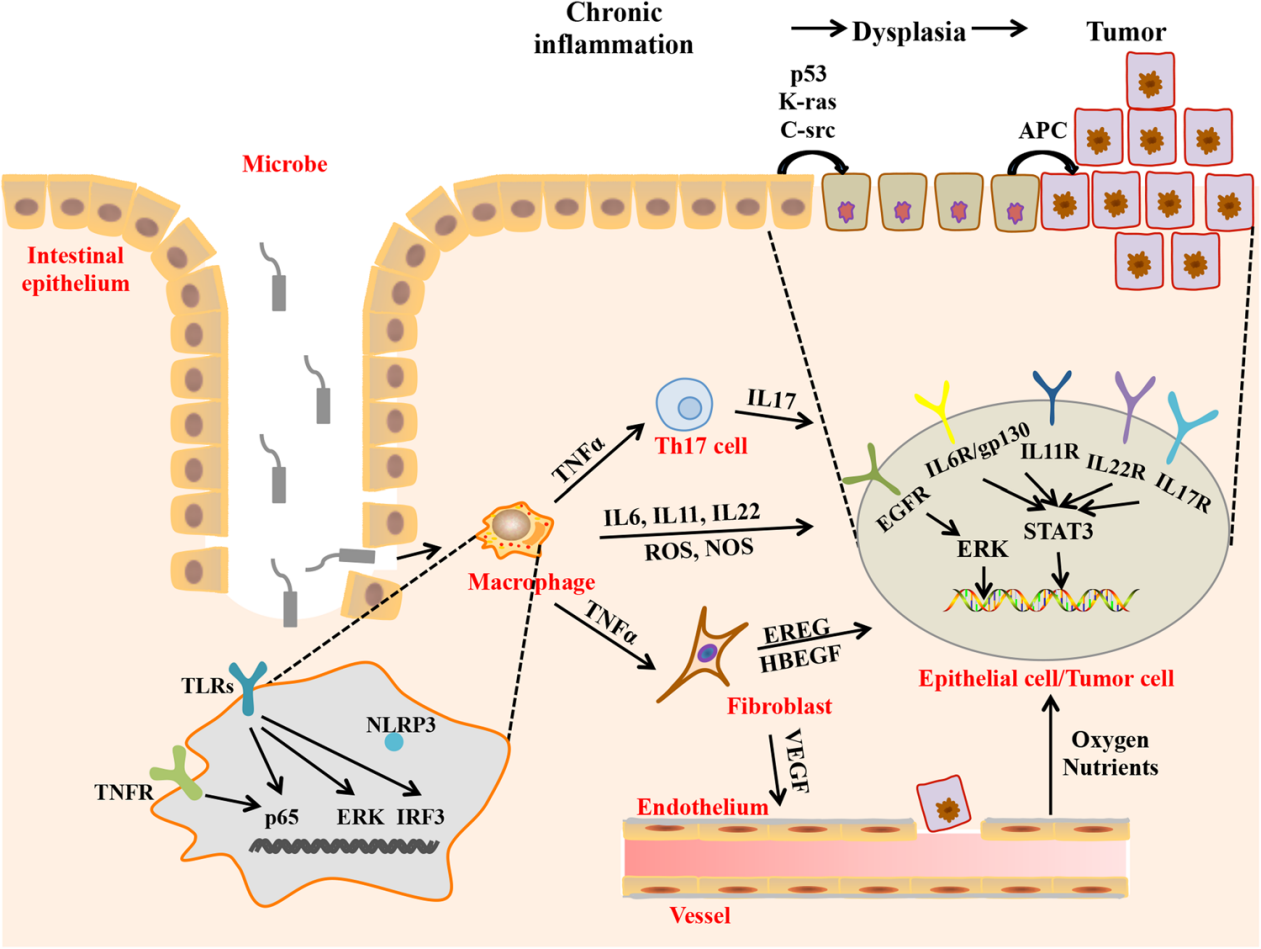
Proposed cellular and molecular mechanism of CAC. Macrophages produce genotoxic substances (ROS and NOS) that cause DNA damage and gene mutation and inflammatory cytokines, such as IL-6, IL-11, and IL-22, which activate STAT3 in intestinal epithelial cells and TNF-α, which activates T cells and fibroblasts. Both T cells and fibroblasts can release cytokines (IL-17 and IL-22, IL-6, EREG, heparin binding EGF like growth factor (HBEGF), and VEGF, respectively). In intestinal epithelial cells or tumor cells, IL-17, IL-22, and IL-6 activate STAT3, and EREG and HBEGF activate ERK. Activation of STAT3 and ERK leads to malignant transformation of epithelial cells. VEGF promotes angiogenesis, which provides oxygen and nutrients for growth and metastasis of cancer cells

The CAC microenvironment is a “complex network” of various cell types, cellular cytokines, signaling molecules, and extracellular matrices that orchestrate the fate of tumorigenesis and tumor development. Immune cells have complex and heterogeneous functions in the CAC microenvironment. Macrophages promote CAC tumorigenesis and progression by producing reactive oxygen species (ROS), nitric oxide synthetase (NOS), and pro-inflammatory cytokines.^5^ Tregs and Th17 cells have tumor-promoting functions during CAC formation,^6,7^ whereas CD8^+^ T cells protect against CAC tumorigenesis.^8^ In addition, cancer-associated fibroblasts (CAFs) promote the growth of colitis-associated neoplasms through activation of ERK signaling and increase colon cancer angiogenesis via IL-6 and VEGF signaling.^9,10^ In addition to providing a survival advantage by inducing angiogenesis, tumor-associated vessels contribute to CAC tumorigenesis by facilitating pro-inflammatory leukocyte extravasation. Therefore, the distinct cell types may be correlated with each other either directly or indirectly and constitute a complex network that regulates the malignant transformation of intestinal epithelial cells as well as CAC formation and progression (Fig. 2).

Recent studies have provided growing evidence of the role of autophagy in inflammation and tumorigenesis.^11^ However, the current data provide conflicting explanations of the mechanisms of autophagy underlying the regulation of CAC tumorigenesis and progression, possibly due to the different types and stages of cancers and particularly, the complicated tumor microenvironment. Therefore, an understanding of autophagy in inflammation and especially during different stages of CAC is certainly anticipated to provide novel opportunities for prevention and therapeutic intervention for CAC. This review will focus on the role of autophagy in the regulation of the tumor microenvironment during different stages of CAC.

## Autophagy in intestinal epithelial cells

The inner lining of the colon consists of an expansive single-cell epithelial layer comprising intestinal epithelial cells (IECs) that exert numerous biological functions, including nutrient absorption, provision of a physical barrier against intestinal toxic substances and secretion of mucus to defend against harmful infection, thereby maintaining intestinal homeostasis. Repeated injury and regeneration of intestinal epithelial cells caused by inflammation or other factors can lead to their malignant transformation and, ultimately, colon cancer. Notably, studies in the past decade have suggested that intestinal epithelial autophagy is strongly involved in and heavily influences each of these processes.

ROS produced during inflammation can induce intestinal epithelial autophagy,^12^ and consequently, intestinal epithelial autophagy reduces excessive ROS levels to protect intestinal epithelia and inhibit intestinal inflammation.^13^ Epithelial autophagy mediated by ATG5 and ATG16L1 contributes to the removal of bacteria from epithelial cells and increases antibacterial defense, leading to a decreased inflammatory response.^14^ Another report suggested that high-mobility group box 1 (HMGB1) protects beclin 1 and ATG5 from calpain-mediated cleavage, which protects epithelial autophagy and reduces tissue injury in IBD.^15^ Additionally, autophagy reduces epithelial tight junction (TJ) permeability via lysosomal degradation of the TJ protein claudin-2,^16^ which may be involved in protecting against intestinal infection and inflammation and promoting epithelial survival by inhibiting mammalian target of rapamycin (mTOR) but activating ERK1/2 and AMP-activated protein kinase (AMPK),^17^ thus supporting intestinal stem cell (ISC) maintenance and regeneration of intestinal epithelial cells.^18^

Indeed, impaired epithelial autophagy is tightly associated with IBD pathogenesis. Colitis patients show reduced levels of ATG5 and ATG16L1.^19^ ATG16L1- and ATG5-deficient Paneth cells exhibit increased peroxisome proliferator-activated receptor (PPAR) signaling and lipid metabolism, which is known to impair intestinal injury responses.^20^ MicroRNAs, including MIR142-3p, MIR106B, and MIR93, can target ATG16L1 to inhibit epithelial autophagy and play a role in intestinal inflammation and CD.^21,22^ Additionally, increased dissemination of invasive bacteria to extraintestinal sites was observed in mice with autophagy-deficient IECs.^23^ Furthermore, reduced autophagy in either DCs or epithelial cells can decrease antigen sampling and increase production of pro-inflammatory-type DCs, which contributes to the colitis process.^24^

Theoretically, epithelial autophagy contributes to the elimination of harmful intestinal microbacteria and ROS, both of which are prone to produce intestinal injury and genomic instability and lead to cancer initiation. As expected, Beclin1^+/–^ mice spontaneously develop various malignancies.^25^ The ras oncogene can promote epithelial malignant transformation through inhibition of the autophagy mediator Beclin-1.^26^ Additionally, atractylenolide I (AT1) can partially activate autophagy through ATG7 to reduce intestinal adenoma initiation.^27^

As discussed above, epithelial autophagy has a prohibitive role in IBD and colorectal tumorigenesis. However, a large number of studies reported that epithelial autophagy promotes cancer development and drug resistance. Autophagy promotes cancer cell proliferation and survival through activation of the AMPK/ULK1 and JAK2/STAT3 pathways while suppressing the expression of p53 and p21.^28–31^ Another study demonstrated that blocking autophagy decreases tumor size via promoting p53 and ER stress-induced apoptosis.^32^ Autophagy reduces radiosensitivity in hypoxic environments through hypoxia inducible factor 1, a subunit (HIF-1α)/miR-210/Bcl-2 pathway in human colon cancer cells.^33^ Inhibition of autophagy by miR-214 promotes radiosensitivity in colorectal cancer.^34^ Oxaliplatin activates cytoprotective autophagy in HT29 xenografts, and inhibition of autophagy by chloroquine (CQ) significantly enhances the anti-cancer activity of oxaliplatin.^35^ In addition, curcumin- or oxaliplatin-induced autophagy is vital for the survival and stemness maintenance of cancer stem cells (CSCs).^36^ Autophagy in colorectal CSCs contributes to paclitaxel chemoresistance.^37^

**Fig. 3:**
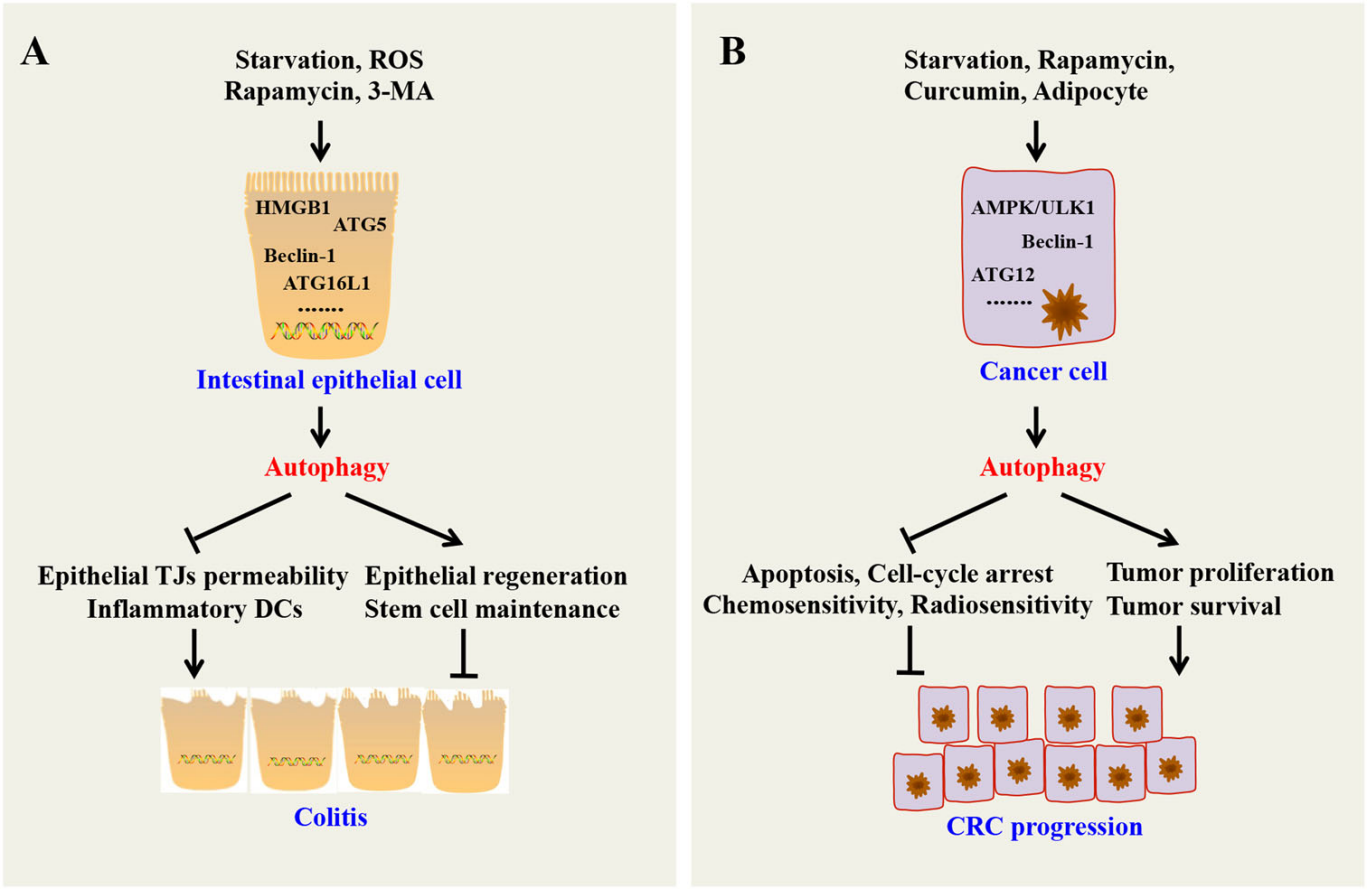
Effects of intestinal epithelial autophagy on colitis and colorectal cancer. **a** Autophagy can suppress colitis through maintaining epithelial TJ permeability; maintaining regeneration of epithelial cells; removing invasive bacteria; and reducing inflammatory cytokines. **b** In established malignant cells, autophagy can accelerate CRC progression through promoting cancer cell proliferation; inhibiting apoptosis and cell cycle arrest of cancer cells; protecting the survival of cancer cells and cancer stem cells in nutrient-deficient conditions; and reducing chemosensitivity and radiosensitivity of cancer cells

Altogether, intestinal epithelial autophagy inhibits colitis and colon cancer initiation but promotes colon cancer development and progression (Fig. 3).

## Autophagy in immune cells

### Autophagy in innate immune cells

**Fig. 4:**
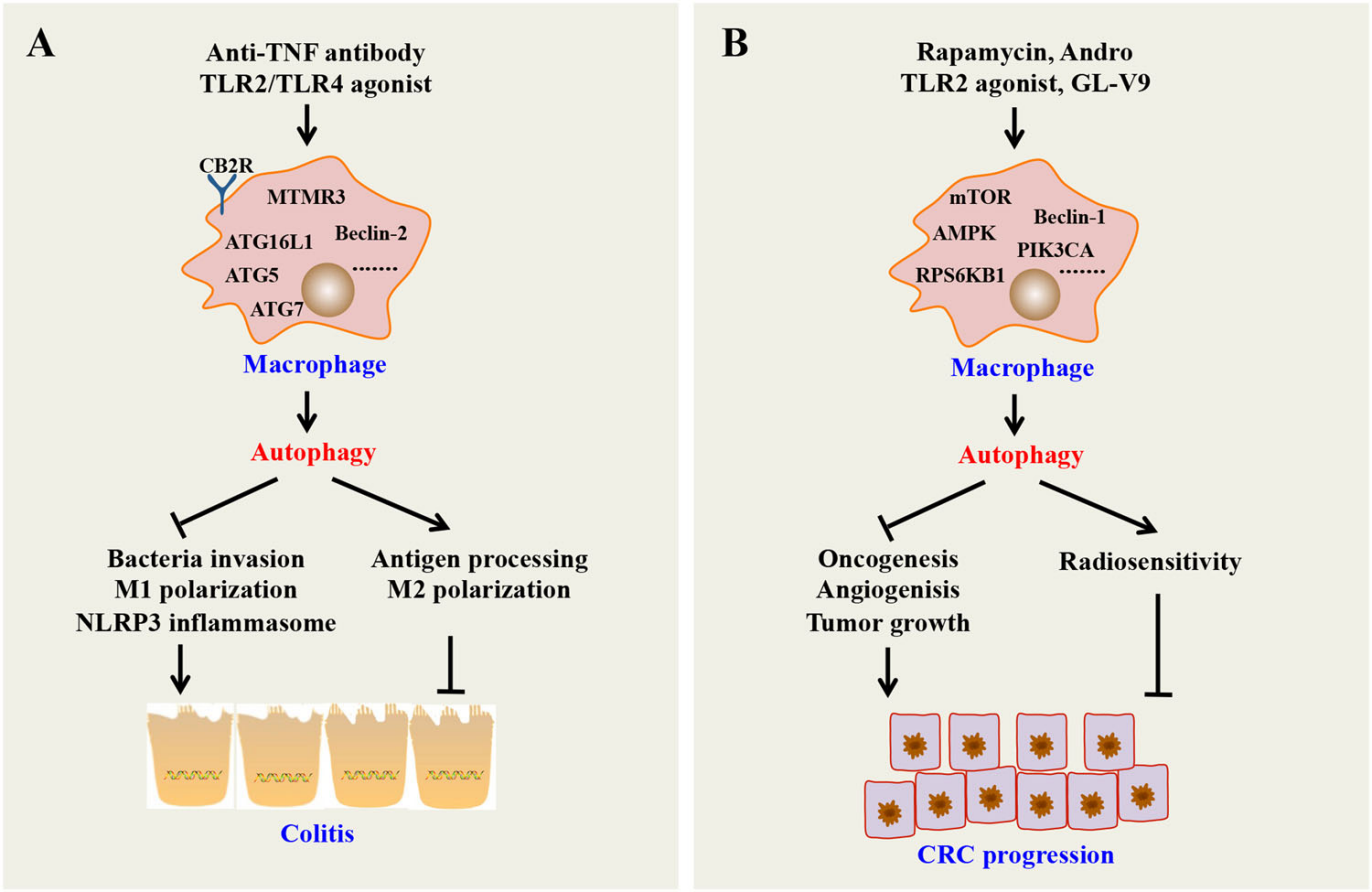
Role of macrophage autophagy on colitis and colorectal cancer. **a** Autophagy can inhibit inflammation through inhibiting bacteria invasion; promoting antigen processing; inhibiting M1 polarization and activating M2 polarization; and suppressing the NLRP3 inflammasome and other PRRs. **b** Autophagy can inhibit CRC through inhibiting oncogenesis, angiogenesis and tumor growth; and increasing the radiosensitivity of CRC cells. MTMR3 myotubularin-related protein 3, CB2R cannabinoid receptor 2

Innate immune cells (such as macrophages, dendritic cells, and neutrophils) can initiate a rapid innate immune response through pattern recognition receptors (PRRs) to regulate host–microbial interactions and cytokine production, which are features of IBD. Macrophage autophagy has been demonstrated to be highly related to regulation of the innate immune response in the gut. For example, promoting autophagy in macrophages inhibits the initiation and activation of the NOD-like receptor family pyrin domain containing 3 (NLRP3) inflammasome, which can trigger maturation of pro-inflammatory cytokines, such as IL-1β and IL-18, upon sensing a wide range of stimuli, thereby suppressing colitis.^38^ However, decreasing autophagy in macrophages can increase PRR-induced IL-1β secretion, nuclear factor of kappa light polypeptide gene enhancer in B-cells (NF-κB) signaling, and ultimately, overall cytokine secretion.^39^ Consistently, mice with myeloid cell-specific deletion of Atg7 were more susceptible to experimental colitis, accompanied by increased colonic cytokine expression and systemic bacterial invasion.^40^ A lack of ATG16L1 in macrophages impairs mitophagy, which increases ROS production, reduces microbial killing, impairs processing of major histocompatibility complex (MHC) class II antigens, and alters intracellular trafficking to lysosomal compartments, leading to colitis development in mice.^41^ Other findings also show that ATG16L1 deficiency alters macrophage function and increases the severity of CD. In addition, loss of autophagy in macrophages has been reported to promote inflammation through increased pro-inflammatory M1 and decreased anti-inflammatory M2 polarization.^42^ Autophagy induced by anti-tumor necrosis factor (TNF) antibodies promotes M2 polarization in macrophages suggesting that macrophage autophagy may have a repressive role in IBD.^43^ Taken together, these findings show that macrophage autophagy can inhibit inflammatory diseases, such as colitis, by regulating inflammatory cytokines and influencing macrophage polarization and microbial killing (Fig. 4a).

Neutrophils function as defenders against pathogens and are one of the essential components of the innate immune system recruited to the site of infection, but abnormal neutrophils may damage gut tissue. Atg7-deficient neutrophil precursors have been shown to exhibit impaired mitochondrial respiration and defective neutrophil differentiation.^44^ Notably, a report indicated that neutrophil autophagy promotes neutrophil-mediated inflammatory responses.^45^ Thus, neutrophil autophagy may heighten colon inflammation.

Another important innate immune cell is the NK cell, which is traditionally considered to be a tumor supervisor and infection defender. Hall et al.^46^ showed that NK cells attenuated DSS-induced colitis and tissue injury by decreasing ROS and cytokine production. However, depletion of NK cells impairs the survival of mice with colitis and dramatically increases colonic damage and inflammation. Wang and colleagues^47^ concluded that autophagy is required for NK cell development and NK cell-induced innate immunity through the removal of damaged mitochondria and intracellular ROS. As it stands, autophagy in NK cells might play an important suppressive role in colitis.

Moreover, autophagy in DCs also plays a vital role in regulating DC function and inflammatory responses. For instance, autophagy-deficient DCs have been associated with lysosomal abnormalities and defective cytoskeletal regulation, with increased cell adhesion and reduced migration, thereby aggravating Crohn’s disease.^48^ In conclusion, autophagy in innate immune cells has an indispensable function in colitis, reflecting the potential of innate immune cells to be used as therapeutic targets in colitis.

In the established cancer microenviroment, tumor-associated macrophages (TAMs) exhibit an M2 phenotype and promote tumor angiogenesis, growth, and metastasis.^49^ However, many studies demonstrated that M1 macrophages inhibit tumor growth.^50^ Recent reports have well established that autophagy plays a crucial role in macrophage production and polarization. For example, Chen et al.^51^ reported that mTOR is critical for macrophage polarization toward the M2 phenotype to promote tumor angiogenesis and growth. TLR2 deficiency significantly suppresses autophagy and leads to an increase in M2 macrophage polarization, which in turn promotes oncogenesis.^52^ The AMPK activator GL-V9 can trigger macrophage autophagy to degrade NLRP3 inflammasomes and protect against colitis and tumorigenesis in mouse CAC.^53^ Another small-molecule andrographolide (Andro) can trigger mitophagy in macrophages to inhibit the NLRP3 inflammasome, which is responsible for CAC prevention.^54^ In addition, upregulation of autophagy in TAMs inhibits proliferation and induces apoptosis in irradiated colon cancer cells, indicating that stimulating TAM autophagy may increase the radiosensitivity of colorectal cancer cells.^55^ Thus, autophagy in TAMs may have a vital role in suppressing cancer. However, it should be noted that some exceptions to this pattern exist. For instance, one study that suggests that autophagy mediated by cathepsin S induces M2-type polarization in TAMs, leading to colon carcinoma development.^56^ Therefore, further studies are needed to assess whether and how these factors affect the function of autophagy modulation in macrophages (Fig. 4b).

Additionally, autophagy of other innate immune cells, such as neutrophils and NK cells, also plays a vital role in the development of colorectal cancer. For instance, tumor-associated neutrophils (TANs) facilitate the initiation and progression of CAC,^57^ and increased neutrophil autophagy is correlated with advanced migration of cancer cells.^58^ Previous reports have suggested that hypoxia-induced autophagy impairs cancer cell susceptibility to NK-mediated apoptosis through selective degradation of the pro-apoptotic protein granzyme B. In vivo data validate the concept that targeting autophagy in cancer cells regresses cancer development by facilitating cancer cell elimination via NK cells.^59^ Similar results have also been observed in melanoma and renal cell carcinoma.^60,61^ DCs are professional antigen-presenting cells of the innate immune system with the potential to generate robust antigen-specific T cell immune responses to prevent tumor development and recurrence.^62^ Importantly, DC autophagy is required for antigen cross-presentation.^63^ In this regard, although further evidence is also needed, autophagy in DCs may inhibit colorectal cancer.

As described above, autophagy in innate immune cells is implicated in colorectal cancer development. The specific role and mechanism of autophagy in the regulation of CRC depends on the microenvironmental innate immune cell types and cancer stages.

### Xenophagy

The gastrointestinal tract hosts a vast array of microbes that are critical for intestinal homeostasis. In CD patients, the numbers and species of microbes are dramatically changed, and certain types of microbes can induce inflammation and promote tumorigenesis, such as *Escherichia coli* and *Fusobacterium nucleatum*.^64^ Additionally, germ-free APC^Min/+^ mice exhibit significantly lower colon tumor incidence and load compared with specific pathogen-free APC^Min/+^ mice.^65^ All of these data suggest that intestinal microbiota play a key role in CAC initiation and development. Accumulating evidence demonstrates that autophagy of pathogens, named xenophagy, is a powerful system of recognizing, capturing and eliminating intracellular bacteria.^66^ Once infected by bacteria, macrophages, neutrophils, DCs and intestinal epithelia can exhibit activated autophagy, thereby killing bacteria, and this activity is vital for the normal intestinal immune response.^67,68^ Among these cells, macrophages are the best studied.

Macrophage xenophagy is mainly mediated by PRRs, such as TLRs and NLRs, which are activated by their pathogen-associated molecular patterns (PAMPs). It has been reported that activation of TLR4 and other TLRs by lipopolysaccharide (LPS), an component of the gram-negative bacterial outer membrane, reduces binding of Beclin 1 by Bcl-2, thereby increasing autophagy in macrophages.^69,70^ In addition, NOD1 and NOD2 activated by NLRs induce autophagy by recruiting Atg16L1, with subsequent sequestration and killing of invading *S. flexneri* and *L. monocytogenes* in autophagosomes.^71^ Following autophagy induction, recognition of invaders as targets is also a vital step in xenophagy. In contrast with nonselective autophagy, xenophagy involves selective degradation of invaders.^72^ For example, the adaptor proteins p62 and NDP52 facilitate selective recognition and autophagy of cytosolic bacteria such as S. typhimurium.^73,74^ All of the above findings suggest that bacteria-induced macrophage autophagy aids in the killing of invasive bacteria, and these findings are further supported by the fact that autophagy deficiency in macrophages leads to bacterial clearance failure.^40^ However, some stubborn intracellular bacteria can disturb the host autophagy pathway by controlling ATG expression, damaging the formation of autophagosome membranes and preventing autophagic recognition.^75^ For example, intracellular Burkholderia cenocepacia significantly reduce the autophagic activity of macrophages by downregulating several ATGs, including ATG9, ATG5, ATG12 and ATG8.^76^ Listeria monocytogenes in macrophage cytosol utilize listeriolysin O (LLO) and phospholipase C (PLC), which damage autophagosome membranes, and actin polymerization protein (ActA), which prevents recognition by the autophagic pathway to allow phagosome escape.^77^

Taken together, autophagy in macrophages plays an important role in the host immune response against invading bacteria, which makes it a potential drug target for anti-colitis and CAC treatment.

### Autophagy in adaptive immune cells

Similar to the innate immune response, the adaptive immune response, especially the cellular immune response, also plays a vital role in the development of inflammation. The adaptive immune system involves recognition of pathogenic or tumor-associated peptides and their presentation on MHC molecules by antigen presenting cells (APCs), including professional APCs, such as DCs, macrophages, B cells, and other nonprofessional APCs. For this purpose, MHC class I molecules are recognized by T cell receptors (TCRs) on CD8^+^ T cells, whereas MHC class II molecules are recognized by TCRs on CD4^+^ T cells. As a result, T cells are activated and differentiate into several types of effector T cells, including Th cells (Th1, Th2, Th17 cells), Tregs and cytotoxic T cells. Th1, Th2, and Th17 cells can produce pro-inflammatory cytokines, such as IL-2, interferon gamma (INF-γ), IL-5, IL-13, and IL-17A, whereas Tregs produce anti-inflammatory cytokines, such as IL-10 and TGF-β. However, cytotoxic T cells release perforin and granzymes, which lead to the apoptosis of infected or malignant cells.^67,78^ Physiologically, the cellular immune response can aid in host defense against pathogenic antigens. However, when T cells are over-activated or the balance between pro-inflammatory and anti-inflammatory T cells is impaired, pathological inflammation, for example, colitis, will occur.

Autophagy has been reported to enhance the adaptive immune response by facilitating APC recognition, MHC-I or MHC-II restricted presentation, and maintaining the survival, function and homeostasis of T cells,^67^ which induces clearance of harmful pathogens. A previous report revealed that DCs utilize autophagic machinery to recognize and present extracellular microbial antigens for MHC-II loading.^79^ The authors found that DCs with defective ATG5 cannot recognize microbes via TLR7 or TLR9 and hence fail to produce IFN-α and present antigens for MHC-II loading in response to these pathogens. In addition, capture and reprocessing by autophagosomes are needed for loading of cytosolic bacteria in DCs onto MHC-I.^80^ T cells with autophagy deficiency, for example, loss of VPS34, have defective mitochondrial function and thus accumulate ROS, which causes an increase in pro-apoptotic protein expression and robust apoptosis of these T cells.^81^ VPS34 ablation in T cells also damages peripheral maintenance and function of Tregs.^82^ Additionally, ATG5 and Beclin 1 deletion result in inefficient proliferation and compromised function of CD8^+^ memory and CD4^+^ T cells, respectively, after TCR stimulation.^83,84^ Conversely, autophagy helps to maintain survival and function of senescent CD8^+^ T cells.^85^

Does autophagy in adaptive immune cells regulate inflammation-associated diseases, such as colitis and CAC? A study conducted by Vrajesh V. Parekh and coworkers has provided a hint to the answer. Their study revealed that Vps34^f/f^;CD4-Cre mice with autophagy-deficient T cells had an increased percentage of CD4^+^ T cells in the spleen and lymph nodes that produced IL-17A and IL-13 but a decreased percentage of Tregs among CD4^+^ T cells in the peripheral lymphoid organs compared with the controls. In addition, through nuclear morphology and cytoplasmic staining, infiltration of inflammatory cells, such as neutrophils and macrophages, in the intestinal lamina propria and inflammatory lesions ranging from hyperplasia to adenoma was observed in Vps34f/f;CD4-Cre mice.^82^ From an immunological point of view, these observations are also in line with the notion that cancer can develop when (pre)malignant cells escape immunosurveillance by actively suppressing antitumor immune responses.^86^

Taken together, autophagy can enhance the adaptive immune response to eliminate pathogenic microbes, regulate T cell homeostasis, maintain the balance between pro-inflammatory T cells and anti-inflammatory T cells and maintain immunosurveillance, which together suppress the development of colitis and even CAC.

## Autophagy in endothelial cells

Blood vessels are lined by endothelial cells in the innermost layer. Formation of new blood vessels is critical for the physiological function of normal tissues and plays a critical role in disease pathology, including cancer. Tumor cell growth and metastasis depend on neovascularization. In clinical practice, chemotherapy plus an angiogenesis inhibitor can result in significant improvement of anti-cancer effect in cancer patients, including the metastatic CRC patients.^87^

**Fig. 5:**
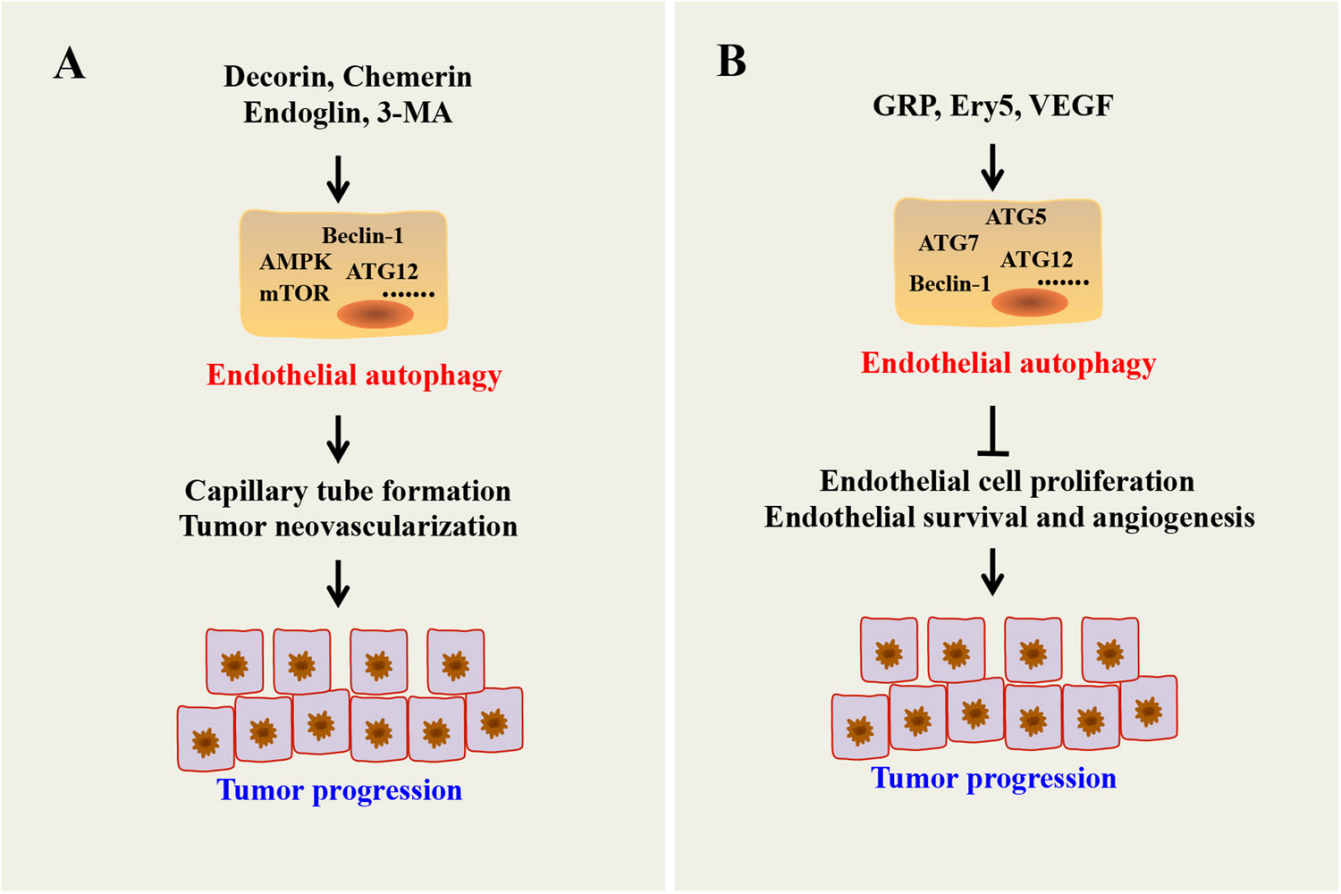
Role of endothelial autophagy on angiogenesis. **a** Autophagy promotes angiogenesis through forming capillary tube. **b** Paradoxically, autophagy suppresses angiogenesis by inducing endothelial cell death and reducing endothelial cell proliferation, migration and tube formation

Accumulating evidence suggests that autophagy plays a vital role in endothelial cell survival, proliferation, migration and angiogenesis. However, whether autophagy plays a positive or negative role in the regulation of angiogenesis is still controversial. For example, Du and coworkers demonstrated that induction of autophagy through overexpression of ATG5 increased bovine aortic endothelial cell (BAEC) tube formation and migration.^88^ Goyal et al.^89^ found that decorin-induced autophagy in endothelial cells had a protective role in tumor neovascularization and epithelial survival. Another report demonstrated that endoglin-mediated autophagy can enhance capillary tube formation.^90^ Indeed, inhibition of autophagy by 3-methyladenine (3-MA) or siRNA against ATG5 reduced angiogenesis.^88^ Inhibition of autophagy by a mitochondria-targeted antioxidant Mito-TEMPO, shRNA against chemerin receptor 23 (ChemR23), AMPKα, or beclin-1 in human aorta endothelial cells (HAECs) impaired chemerin-induced tube formation and cell proliferation.^91^ Paradoxically, Ery5 (a derivative of the natural product magnolol)-induced autophagy effectively inhibited cell proliferation, migration, invasion and tube formation.^92^ Autocrine VEGF from endothelial cells and gastrin-releasing peptide (GRP) secreted by tumors are necessary for endothelial cell proliferation, endothelial survival and angiogenesis through autophagy inhibition and autophagic cell death of endothelial cells.^93^ Additionally, a study conducted by Seon-Jin Lee and colleagues demonstrated that heterozygous disruption of Beclin1 accelerates tumor growth and increases angiogenesis under hypoxia^94^ (Fig. 5a, b).

Although controversy exists, several studies have reported that combination therapy with an autophagy inhibitor and an angiogenesis inhibitor can exert enhanced antiangiogenic effects and have a potential antitumor effect. For instance, kringle 5 (K5) of human plasminogen, a potent angiogenesis inhibitor, can induce autophagy and apoptosis in endothelial cells. Knockdown of Beclin 1 via RNA interference decreased K5-induced autophagy but accelerated K5-induced apoptotic cell death, which suggests that interfering with the autophagic survival response can potentiate the antiangiogenic effects of K5 in endothelial cells, leading to a potential antitumor effect.^95^ Similarly, sulforaphane (SUL), an isothiocyanate, has pro-apoptotic and pro-autophagy effects on tumor vascular endothelial cells. Co-treatment of HAECs with SUL and the autophagy inhibitor 3-MA enhanced the pro-apoptotic effect but weakened the ability of HAECs to form capillary-like structures, which suggests the possibility of using autophagy inhibitors in combination with anti-angiogenic agents to treat cancer.^96^

Overall, autophagy can regulate the process of angiogenesis, which is important for tumor growth, by influencing the survival and function of endothelial cells, thereby exerting a pro- or anti-tumor effect on CAC.

## Autophagy in mesenchymal cells

Fibroblasts are embedded within the fibrillar matrix and are a principal cellular component of the connective tissue. The important functions of fibroblasts include deposition of extracellular matrix (ECM), regulation of epithelial cell differentiation, regulation of inflammation, and involvement in wound healing. Abnormally activated fibroblasts are also involved in the development of inflammatory disease and cancer. Fibroblasts help define an additional stromal address that directs leukocyte behavior within tissues. Chronic inflammation is characterized by the abnormal persistence of inflammatory cell infiltration, as well as a local increase and activation of fibroblasts in the disease nidus.^97^ In addition, a series of studies have indicated that tumor growth is not just determined by malignant cancer cells themselves but also by their surrounding stromal microenvironment.^98^ In addition to endothelial cells and inflammatory cells, it is becoming increasingly clear that fibroblasts in cancer, so-called CAFs, can promote tumor growth, progression, and metastasis.

Autophagy is an important pro-survival regulator of fibroblast growth. For example, inhibiting autophagy in fibroblasts via administration of siRNAs targeting ATG5 and beclin 1 or 3-MA and bafilomycin A1 blocked IL-2-induced autophagy, followed by inhibition of IL-2-induced fibroblast proliferation and enhancement of apoptosis.^99^ In addition, inflammation can induce fibroblast autophagy. Human gingival fibroblasts treated with LPS from Porphyromonas gingivalis exhibited increase expression of ATG12 and LC3-1.^100^ A study reported that chemotherapies could transform stromal fibroblasts into CAFs phenotypically and metabolically, leading to the emergence of a highly autophagic microenvironment and in turn activating the stemness of adjacent epithelial cells, which can potentially trigger tumorigenesis.^101^ Additionally, Capparelli C and colleagues demonstrated that overexpression of the autophagy genes, BCL2 interacting protein 3 (BNIP3), cathepsin B (CTSB) or ATG16L1 in CAFs to induce an autophagic phenotype with features of mitophagy and mitochondrial dysfunction can induce the production of high-energy mitochondrial fuels, such as L-lactate, ketone bodies, glutamine and free fatty acids, which promote tumor growth and metastasis.^102^ Indeed, inhibition of autophagy in colorectal CAFs can affect colorectal cancer development and increase the radiosensitivity of colorectal cancer.^103^

Above all, fibroblast autophagy is important for fibroblast growth and enhances the interaction between fibroblasts and cancer cells, which may promote the initiation and progression of colon inflammation and cancer.

## Therapeutic potential of autophagy modulators in the treatment of colitis and colorectal cancer

Because colitis and colorectal cancer are diseases involving several distinct stages and multiple microenvironmental cells, autophagy fulfills a dual role, having colitis or CRC-promoting and colitis or CRC-suppressing properties. Functional autophagy prevents necrosis and inflammation, which can lead to genetic instability. On the other hand, autophagy might be important for tumor progression by providing energy through its recycling mechanism during unfavorable metabolic circumstances. Although the exact role of autophagy in the development of colitis and CRC remains controversial, and how autophagy should be manipulated when treating patients is not fully defined, there are a number of effective pharmacologic mediators of autophagy in preclinical and clinical use today (Table 1).^35,54,104–119^

**Table 1:** Examples of autophagy inhibitors and activators for colitis/CRC therapy

Drugs	Mechanism	Effects	Ref.
Autophagy inhibitors			
3-MA	Prevents autophagosome formation	Enhances the apoptosis effect of 5-FU (5-fluorouracil) in CRC	^104^
CQ	Inhibits lysosomal acidification and prevents fusion with autophagosomes	1. Enhances the apoptosis effect of 5-FU in CRC	^35, 105– 108^
		2. Augments sunitinib-induced apoptosis and enhances the antiangiogenic capacity of sunitinib	
		3. Enhances trichostatin A-induced apoptosis in radiotherapy-treated colon cancer cells	
		4. Enhances sensitivity to oxaliplatin and bevacizumab	
		5. Triggers apoptosis of CSCs and decreases colonosphere formation ability combined with PDT (photodynamic therapy) in vitro and tumorigenicity in vivo	
Lys05	Deacidifies the lysosome	Displays single-agent antitumor activity in vivo	^109^
Bafilomycin-A1	Inhibits fusion between autophagosomes and lysosomes	Enhances pyrrolo-1,5-benzoxazepine-6-induced apoptosis	^110^
Vitexin	Downregulates ATG5, beclin-1, LC3-II	Induces apoptosis and suppresses tumor growth in an HCT116 xenograft model	^111^
Autophagy activators			
Andrographolide	Suppresses the PIK3CA-AKT1-MTOR-RPS6KB1 pathway	Attenuates colitis progression and tumor burden	^54^
AZD-2014	Inhibits mTOR	Inhibits the growth of HT-29 cell xenografts in SCID mice and improves mice survival	^112^
Silibinin	Induces autophagic death mediated by endoplasmic reticulum stress	Induces autophagic death of colon cancer cells and CRC xenograft	^113^
Temsirolimus	Inhibits mTOR	Inhibits cell growth via anti-angiogenesis activity and enhances apoptosis	^114^
Resveratrol	Induces autophagy-mediated through ROS	Induces apoptosis	^115^
Bufalin	Induces autophagy-mediated through ROS	Induces autophagic cell death	^116^
BIX-01294	Induces autophagy via EHMT2 (euchromatic histone-lysine N-methyltransferase 2) dysfunction and intracellular ROS accumulation	Induces autophagy cell death	^117^
Celastrol	Suppresses the PI3K/Akt/mTOR signaling pathway	Ameliorates experimental colitis in IL-10-deficient mice	^118^
BCG/CWS (Mycobacterium bovis Bacille Calmette–Guerin cell wall skeleton)	Induces autophagy-mediated through ROS	Enhances radiotherapy effect in colon cancer cells	^119^

## Conclusions and perspectives

Autophagy plays a significant role in the initiation and progression of CAC. The exact function of autophagy in CAC is dependent on the CAC stage and its microenvironmental context. For example, intestinal epithelial autophagy can eliminate unnecessary products, such as damaged proteins and organelles, ROS or harmful microbacteria, thereby suppressing CAC initiation by repressing inflammation and stabilizing the genome. However, cytoprotective autophagy in colon cancer cells plays a tumor-promoting role and leads to chemotherapy and radiotherapy resistance in established CAC. Furthermore, autophagy in other cells, such as immune cells (macrophages, neutrophils, NK cells, DCs, T cells), endothelial cells and mesenchymal cells, which constitute the colon cancer microenvironment, also has a different but strong influence on CAC development. Therefore, we can draw the following conclusion: autophagy subtly controls CAC according to the different stages and tumorous microenvironment (Tables 2 and 3).

**Table 2:** Role of autophagy in IBD

Cell	The role of autophagy in IBD	Mechanism	Ref.
Epithelial cell	Inhibits colitis	Inhibits ROS, removes bacteria, reduces epithelial TJ permeability, and supports ISC maintenance and regeneration of epithelial cells	^12– 18, 22, 23^
Macrophage	Inhibits colitis	Inhibits PRR-induced ROS, cytokines and NLRP3 inflammasomes	^38– 40^
Neutrophil	Promotes colitis	Produces NADPH-oxidase-mediated reactive oxygen species	^45^
NK cell	Protects innate immunity	Removes damaged mitochondria and intracellular ROS	^47^
DC	Inhibits colitis	Decreases inflammation and Th17 responses	^48^
T cell	Inhibits colitis	Decreases CD4^+^T cells that produce IL-17A and IL-13 and CD8^+^T cells that produce IFN-γ; increases Tregs	^86^
Endothelial cell	NA		
Mesenchymal cell	NA

**Table 3:** Role of autophagy in CRC

Cell	The role of autophagy in CAC	Mechanism	Ref.
Epithelial cell	Inhibits tumorigenesis	Eliminates harmful intestinal microbacteria and ROS	^25– 27^
	Promotes development of CAC and resistance to chemotherapy	Promotes cancer cell proliferation and survival and protects the stemness and chemoresistance of colorectal CSCs	^28– 37^
Intestinal macrophage	Inhibits tumorigenesis	Degrades NLRP3 and inhibits M2 polarization	^52– 54^
Tumor-associated macrophage	Controversial	Increases radiosensitivity by inducing colon cancer cell apoptosis or promotes colon cancer development by inducing M2-type polarization of TAMs	^55, 56^
Neutrophil	Promotes tumor migration	Produces pro-metastatic oncostatin M and MMP9	^58^
NK cell	NA		
DC	NA		
T cell	Inhibits tumorigenesis	Decreases CD4^+^T cells that produce IL-17A and IL-13 and CD8^+^T cells that produce IFN-γ; increases Tregs	^86^
Endothelial cell	Promotes or inhibits tumor neovascularization	Depends on the autophagy molecules and microenvironment	^88– 96^
Mesenchymal cell	Promotes tumorigenesis and cancer development	Produces high-energy mitochondrial fuels	^101– 103^

CAC includes several stages and is regulated by many tumorous microenvironmental cells. There is an immune dialogue between IEC/CRC cells and immune cells. For example, immune cells produce inflammatory cytokines that play a crucial role in EIC inflammation and tumorigenesis. Tumor cells may over-express pro-inflammatory mediators, which in turn activate immune cells for inflammatory cytokine production. Endothelial cells are not only involved in neovascularization but also participate in tumor immune response.^120^ In addition, there are complicated links between innate and adaptive immunity. Autophagy is involved in every stage and in all the microenvironmental CAC cells. Therefore, it is not sufficient to target autophagy in a certain node to treat CAC, taking the entire network system into consideration is needed and helpful. It is necessary to establish a precise research model and explore the explicit role of autophagy in this network. For example, knockout some key autophagy regulators in specific tissues and in specific stages. Some reports have demonstrated that an autophagy inhibitor or activator combined with chemotherapy or radiotherapy exhibits an enhanced therapeutic effect on CAC, but several aspects should be taken into consideration. For example, which autophagy-related molecule is the most suitable target? What is the most appropriate intervention time point? How should autophagy regulators be delivered with more specific targeted materials in mouse CAC models? Considering the complicated role of autophagy in CAC, it will take a long time to translate scientific research into clinical practice.
